# On the Adaptive Partition Approach to the Detection of Multiple Change-Points

**DOI:** 10.1371/journal.pone.0019754

**Published:** 2011-05-24

**Authors:** Yinglei Lai

**Affiliations:** Department of Statistics and Biostatistics Center, The George Washington University, Washington, D.C., United States of America; University of Swansea, United Kingdom

## Abstract

With an adaptive partition procedure, we can partition a “time course” into consecutive non-overlapped intervals such that the population means/proportions of the observations in two adjacent intervals are significantly different at a given level 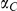. However, the widely used recursive combination or partition procedures do not guarantee a global optimization. We propose a modified dynamic programming algorithm to achieve a global optimization. Our method can provide consistent estimation results. In a comprehensive simulation study, our method shows an improved performance when it is compared to the recursive combination/partition procedures. In practice, 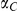 can be determined based on a cross-validation procedure. As an application, we consider the well-known Pima Indian Diabetes data. We explore the relationship among the diabetes risk and several important variables including the plasma glucose concentration, body mass index and age.

## Introduction

Time-course data analysis can be common in biomedical studies. A “time course” is not necessarily only a certain period of time in the study. More generally, it can be patients' age records or biomarkers' chromosomal locations. A general “time” variable can be a predictor with continuous or ordinal values. When we analyze a response variable (binary, continuous, etc.), it is usually necessary to incorporate the information from this predictor. Many well-developed regression models can be used for the analysis of this type of data. In this study, we focus on a nonparametric type of analysis of time-course data: the whole time course is partitioned into consecutive non-overlapped intervals such that the response observations are similar in the same block but different in adjacent blocks. Then, the partition of time course is actually the detection of change-points. The detection of a single change-point has been well studied in statistical literature [1]. However, for the detection of multiple change-points, since there are many unknown parameters like the number of change-points, the locations of change-points and the population means/proportions in each block, it still remains a difficult problem [2].

A motivating example for this study is described as follows. The body mass index (BMI) is calculated by dividing the mass (in kilograms) by the square of the height (in meters). The recent WHO classification of BMI gives six categories: underweight (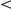
18.5), normal weight (18.5-24.9), overweight (25.0–29.9), class I obesity (30.0–34.9), class II obesity (35.0–39.9) and class III obesity (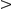
40.0). For the continuous variable BMI, its values are classified into six categories based on five cut-off points. Normal weight is considered as low risk, while the risk of underweight category is elevated, and the risks of overweight, class I, II and III obesity are gradually increased. Therefore, as BMI increases, the risk trend is not simply increasing nor decreasing, but is a “U” shape. A question motivated from this classification is that, given the data of BMI and health status, can we partition the variable BMI into consecutive non-overlapped intervals (categories) such that the risks are similar within an interval but significantly different between two adjacent intervals?

In this study, our purpose is to partition a “time course” into consecutive non-overlapped intervals such that the population means/proportions of the observations in two adjacent intervals are significantly different at a given level 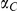. This type of analysis can provide informative results in practice. For example, medical experts may provide an appropriate consultation based on a patient's blood pressure level.

The isotonic/monotonic regression (or the order restricted hypothesis testing) is a traditional nonparametric trend analysis of time-course data [3]. Since the maximum likelihood estimation results are increasing/decreasing piecewise constants over the time course, this analysis can also be considered as a special case of change-point problem. Based on the traditional isotonic/monotonic regression, the reduced isotonic/monotonic regression has been proposed so that the estimation results can be further simplified [4]. Its additional requirement is that the estimated population means in two adjacent blocks must be significantly different at a given level. However, the existing method is based on a backward elimination procedure and does not guarantee the maximum likelihood estimation results.

Without the constraint of trend shape, the detection of multiple change-points for our study purpose can be achieved through a recursive algorithm based method like recursive combination or recursive partition. The circular binary segmentation algorithm is a typical example of recursive partition [5]. In the middle of a large block, the method recursively tries to detect a sub-block with a significantly different population mean. The analysis results are piecewise constants. This method has been frequently used for analyzing array-CGH data [6]. The reduced isotonic regression (see above) is an example of recursive combination. These recursive algorithms provide approximated solutions as alternatives to the globally optimized solutions since an exhaustive search is usually not feasible. Therefore, global optimizations are not always guaranteed.

The dynamic programming algorithm is a frequently used method for optimizing an objective function with ordered observations [7]. Therefore, it is intuitive to consider this algorithm in the analysis of time-course data. This algorithm has actually been frequently used to implement many statistical and computational methods [8] [9] [10] [11] [12] [13]. For a feasible implementation of this algorithm, an optimal sub-structure is necessary for the objective function. This is usually the case for the likelihood based estimation in an unrestricted parameter space. However, when there are restrictions for the parameters, certain modifications are necessary for the implementation of the dynamic programming algorithm.

In the following sections, we first present a modified dynamic programming algorithm so that a global optimization can be achieved for our analysis. The algorithm has been originally developed for the normal response variables. But the extension of our method to the binary response variable is straightforward and is also discussed later. We prove that this method can provide consistent estimation results. Then, we suggest a permutation procedure for the 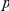-value calculation and a bootstrap procedure for the construction of time-point-wise confidence intervals. We use simulated data to compare our method to the recursive combination/partition procedures. The well-known Pima Indian Diabetes data set is considered as an application of our method. We explore the relationship among the diabetes risk and several important variables including the plasma glucose concentration (in an oral glucose tolerance test, or OGTT), body mass index (BMI) and age.

## Methods

### A modified dynamic programming

At the beginning, we introduce some necessary mathematical notations. Consider a simple data set with two variables: 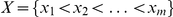 represents 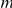 distinct ordered indices (referred to as “time points” thereafter), and 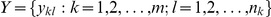 represents the observations with 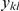 being the 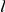-th observation at the 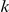-th time point. Let 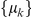 be the corresponding population mean of 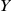 at the 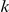-th time point. We assume that 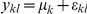, where 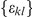 are independently and identically distributed (i.i.d.) with the normal distribution 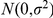. Furthermore, we assume that the set 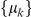 has a structure 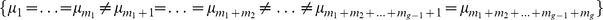 with 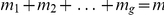. 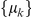, 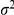 and the set 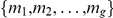 are all unknown (including 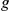) and to be estimated.

The traditional change-point problem assumes that 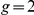. When 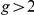, it is a multiple change-point problem. If there is no strong evidence of change points, we may consider the null hypothesis of no change-point (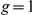) 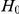: 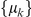 are the same. For the traditional analysis of variance (ANOVA), we consider an alternative hypothesis 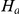: 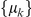 can be different. Then, even when the null hypothesis 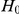 can be significantly rejected, there may be many adjacent 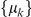 estimated with similar values. Therefore, we intend to group similar and adjacent 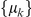 into a block. If this is achievable, then we can have a detection of multiple change-points. Therefore, we specify the following restricted parameter space for the alternative hypothesis:


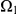
: 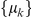 can be different; if we claim any 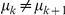, then they are significantly different at level 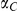 by a two-tailed (or upper-tailed/lower-tailed) test with the two-sample data partitioned to include 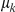 and 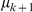 in each sample.

#### Remark 1

The comparison based on adjacent time points has no effect and 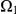 is reduced to 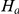 when 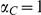. Clearly, 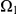 is reduced to 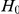 when 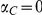. Furthermore, when an upper-tailed/lower-tailed test is specified, the analysis is the reduced isotonic/monotonic regression [4]. Particularly, the analysis is the traditional isotonic/monotonic regression [3] when 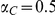 (when a one-sided 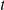-test is used for comparing two sample groups).

The goal of this study is to partition 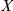 into consecutive non-overlapped intervals such that the population means of the observations in two adjacent intervals are significantly different at a given level 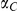. This type of analysis cannot be achieved by the computational methods for the order restricted hypothesis testing (or the isotonic regression) due to the existence of significance parameter. One may consider the well-known dynamic programming (DP) algorithm [7] since the observations are collected at consecutive time points. This is again not feasible: an optimized partition for a subset of time points may be excluded for a larger set of time points due to the significance requirement in 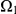. However, we realize that the traditional DP algorithm can be modified to achieve our goal by adding an additional screening step at each time point.

#### Algorithm

Due to its satisfactory statistical properties, the likelihood ratio based test (LRT) has been widely used. To conduct a LRT, we need to estimate the parameters under different hypotheses. When normal population distributions (with a common variance) are assumed for 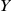, the maximum likelihood estimation is equivalent to the estimation by minimizing the sum of squared errors (SSE). When a block of time points 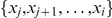 are given, the associated SSE is simply 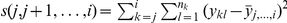, where 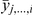 is the sample mean of the observations in the time block 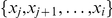. Notice that the SSE of several blocks is simply the sum of SSEs of individual blocks. Then, under the alternative hypothesis, we propose the following algorithm that is modified based on the well-known DP algorithm. For simplicity, we refer to the term “triplet” as a vector containing (link, index, score) that are described in the algorithm. (For each triplet, “link” is defined as the time point right before the block under current consideration; “index” is defined as the index in the triplet set linked from the time point under current consideration; “score” is defined as the objective function value under current consideration.)

link 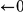; index 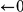; score 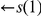


Create 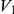 as a vector set with only one element with the above triplet

**for** 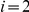 to 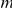 **do** 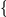


link 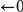; index 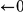; score 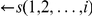


Create 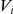 as a vector set with only one element with the above triplet

**for** 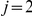 to 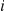 **do** 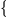


Go through 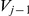 as ordered until a feasible index can be found 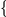


link 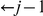


index 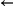 current position in 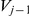


score 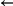 (current score in 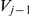) + 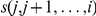


Include the above triplet as a new element in 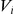 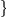 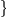


Sort 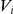 according to the increasing order of SSE scores
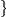


#### Remark 2

It is important that the maximum likelihood estimation is equivalent to the estimation by minimizing the sum of squared errors (SSE) when normal population distributions (with a common variance) are assumed for 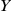. Notice that a normal distribution is assumed for each time point. The population means can be different at different time points but the population variances are common for all the time points. Due to the optimal substructure requirement for the dynamic programming algorithm, we can only estimate the parameters specific to the existing partitioned blocks. As shown in the above algorithm, the estimation of variance can be achieved after the estimation of population means. (The algorithm will not work if the common population variance has to be estimated within the algorithm.)

#### Remark 3

**Figure 1:**
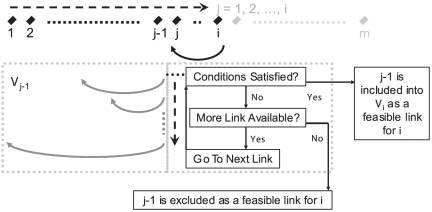
An illustrative flow chat for the modified dynamic programming algorithm.

The definition for a feasible index in 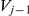 is a time point 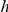 such that two population means in the blocks 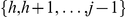 and 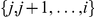 are significantly different at level 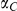 (as specified in the restricted parameter space 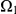). A flow chat is given in Figure 1 to illustrate this algorithm. The set 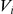 contains an optimized link index among the feasible ones for each of its previous time point 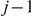, 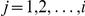 (if there is no feasible link index for a previous time point 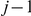, then 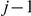 will be excluded from 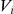). Since the required condition for the restricted population means are imposed on adjacent blocks, the set 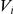 also contains all the necessary link point for the future time points when the time point 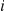 is screened as a link point. (Then, it is not necessary to check other time points 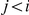 not included in 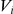.) This can be confirmed as follows: if any future time point uses the time point 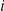 as a link point, then any sub-partitions stopped at the time point 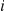 must meet the required conditions for the restricted population means; furthermore, an optimized one will be chosen from the feasible ones for each time point before the time point 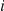; therefore, these sub-partitions belong to 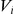.

#### Theorem 1

With the mathematical assumptions described at the beginning, the proposed modified dynamic programming algorithm solves the maximum likelihood estimation of restricted population means.

**Proof:** With the above discussion, it is not difficult to give a proof. It is obvious to prove this claim at the time points 1 and 2 since the algorithm just enumerates all possible partitions and selects the optimized one. Assume that the claim holds at the time point 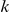. For the time point 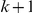, the algorithm screens all the previous time points and selects the optimized and feasible solution (when it exists) for each of them. Finally, all these locally optimized solutions are sorted so that a global optimized solution can be found. Therefore, the claim also holds at the time point 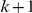. This concludes the proof that the claim holds for all the time points.

#### Remark 4

To test whether 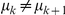 (or 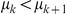 or 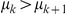) is significant at level 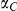, we consider the well-known two-sample Student's 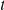-test. The statistical significance (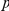-value) of a test value can be evaluated based on the theoretical 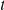-distribution or through the permutation procedure [14]. Considering the likely small sample size on each time point and the computing burden involved in this analysis, the theoretical 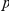-value may be a more preferred choice when the observations are approximately normally distributed. (The required sample size for calculating a 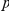-value is no less than two for each sample; otherwise, one will be considered as the reported 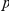-value.)

#### Estimators

When we finish screening the last time points, the overall optimized partition can be obtained in a backward manner:
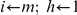
Create the set 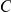 with an element 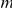


**while** 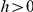 **do** 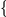 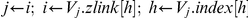
Include 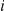 as a new element in the set 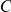 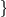


The estimated means 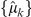 in the restricted parameter space 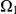 are simply the sample means for the partitioned blocks. With 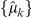 calculated, we can estimate the variance by 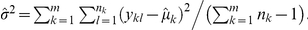


Compared to the traditional dynamic programming algorithm, which requires 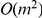 computing time, our modified algorithm requires at least 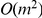 but at most 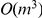 computing time. The additional computing time is necessary so that the optimization can be achieved in the restricted parameter space 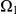.

### Consistency of Estimation

The following theorem shows that our proposed algorithm can provide consistent estimates for 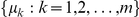 and 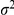. The mathematical proof is given in File S1.

#### Theorem 2

Let 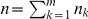. Assume that 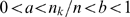 and 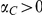. Then, for any time point 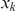, we have 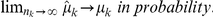 Furthermore, we also have 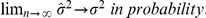


Here, we briefly provide an outline of proof for the readers who wish to skip the mathematical derivation. When the sample size at each time point becomes larger and larger, eventually the true structure 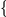 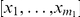, 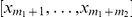, 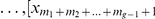, 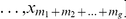 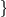 will be a feasible partition of time points (since the power of the two-sample tests for these adjacent partitions will go to 100%). Its corresponding estimates are actually the sample means and they are consistent estimators. Furthermore, the estimated variance, which is closely related to the SSE, will be eventually optimal when the sample size becomes larger and larger. However, our algorithm guarantees a minimized SSE. Then, the estimated population means provided by our algorithm will be closer and closer to the underlying sample means. Therefore, our algorithm can provide consistent estimates for the underlying population means. Then, it is straightforward to prove the convergence of the variance estimator.

#### Remark 5

The estimation bias and variance for isotonic regression are difficult problems [15] [16] [17]. These two issues are even more difficult for our adaptive partition approach since a two-sample test is involved in the detection of multiple change-points. (However, the building-in two-sample test can be an appealing feature for practitioners.) Therefore, we use the well-known permutation and bootstrap procedures to obtain the 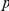-value of test and the confidence limits of estimates. They are briefly described as below.

### 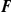-type test and its 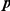-value

We use 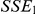 to denote the score in the first element of 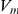. This is the optimized SSE associated with the restricted parameter space 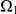. The SSE associated with the null hypothesis is simply 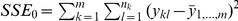. Then, we can define a 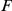-type test:
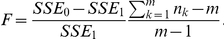
It is straightforward to show that 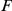 is actually a likelihood ratio test. However, it is difficult to derive the null distribution of 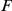 due to the complexity of our algorithm. Therefore, we propose the following permutation procedure for generating an empirical null distribution.

1. Generate 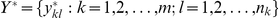 as a random sample (without replacement) from 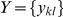;
2. Run the modified DP algorithm with 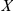 and 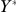 as input and calculate the associated 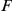-type test 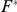;
3. Repeat steps 1&2 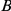 times to obtain a collection of permuted 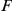-scores 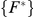, which can be considered as an empirical null distribution.

The procedure essentially breaks the association between 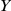 and 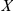. It is also equivalent to permute the expanded time point set 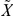 (see below). In this way, the null hypothesis can be simulated with the observed data and the null distribution of 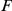 can be approximated after many permutations [18]. Then, the 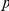-value of an observed 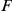-score can be computed as: (number of 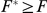)/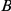. For a conservative strategy, we can include the observed 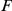 into the set of permuted 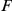-scores (since the original order is also a permutation). We can use this strategy to avoid zero 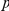-values.

### Time-point-wise confidence intervals

Our algorithm provides an estimate of population mean at each time point. It is difficult to derive the theoretical formula for constructing a confidence interval. Instead, we can consider a bootstrap procedure. At the beginning, we need to expand the variable 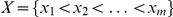 to 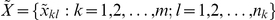, where 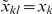 for 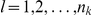. Then, we denote 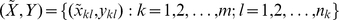.

1. Generate 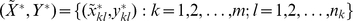, where 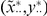 is re-sampled based on 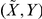 with replacement;
2. Run the modified DP algorithm with 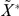 and 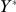 and estimate the time-point-wise population means; if any time points in 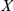 is not re-sampled in 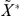, then assign missing values as the estimates for those time points;
3. Repeat steps 1&2 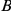 times to obtain a collection of resample estimates of time-point-wise population means 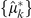.

The procedure applies the “plug-in principle” so that a resample distribution can be generated for each time point [19]. A pair of empirical percentiles (e.g. 2.5% and 97.5%) can be used to constructed a confidence interval for each time point after excluding the missing values.

### Extension to binary response variables

It is straightforward to extend our method for the binary response variables. This can be simply achieved by changing the objective function (SSE in our current algorithm) to the corresponding (negative) log-likelihood function for the binary response variable, and also changing the two-sample 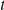-test in our current algorithm to the corresponding two-sample comparison test for the binary response variable. Due to the computing burden, we would suggest to use the two-sample 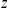-test for proportions when there is a satisfactory sample size or the Fisher's exact test when the sample size is small (e.g. less than six in each cell of the 2×2 contingency table).

### Choice of 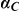


An appropriate choice of 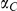 is important. A small number of partitioned blocks will be obtained if a small value is set for 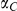, and vice versa. For examples, no partition will be obtained if 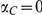 and each time point will be a partition if 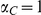. Therefore, like the smoother span parameter for the local regression [20], 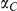 can also be considered as a smoothing parameter. In practice, we suggest to use the cross-validation procedure [21] to select 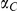. Among a given finite set of values like 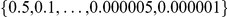, we can choose the one that minimizes the prediction error.

### Approximation algorithms: recursive combination and recursive partition

To illustrate the advantage of global optimization in the likelihood estimation of restricted population means, we also consider and implement two widely used approximation algorithms: recursive combination and recursive partition. These algorithms provide approximated (sometimes exact) solutions to the optimal solution in the restricted parameter space defined based on the given 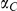. Based on the following description of these two algorithm, it is clear their required computing time is at most 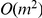.

For the recursive combination algorithm, it begins with no partition and each time point is a block. In each loop, it conducts a two-sample test for each pair of adjacent blocks and find these pairs with 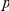-value higher than 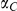; among the combinations based on these selected pairs of adjacent blocks, the one that results the largest overall likelihood (based on all the data) is chosen and the next loop is started when it is still possible to combine the existing blocks; otherwise, the algorithm stops and returns the partitioned blocks. [Notice that this algorithm is slightly different from the one proposed by [4], in which the pair of blocks is chosen completely based on the two-sample test. In our simulation studies, we have observed that the likelihood based criterion can result in a better performance (results not shown).]

For the recursive partition algorithm, it begins with one block with all the time points. In each loop, within each existing partitioned block, it conducts a two-sample test for each possible partition (that generates two smaller blocks) and find these triplets (when the partitions are from the blocks in the middle of time course) or pairs (when the partitions are from the blocks on the boundaries of time course) with test 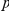-values lower than 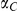; among the partitions based on these selected triplets/pairs, the one that results the largest overall likelihood (based on all the data) is chosen and the next loop is started when it is still possible to create new partitions; otherwise, the algorithm stops and returns the partitioned blocks.

### Performance evaluation

In a cross-validation (CV) procedure (e.g. leave-one-out or 10-fold CV), the estimated population mean 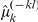 for each observation 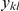 can be obtained based on the training data without 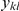. (If the time point 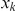 is not included in the training set, then 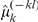 can still be obtained based on the linear interpolation between two nearest time points to 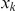.) Then, the CV (prediction) error is calculated as 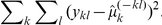


#### Remark 6

In a simulation study, instead of a CV error, we can use the overall mean squared error since we know the parameter values. This is a strategy to save a significant amount of computing time. For each round of simulation, the overall mean squared error is calculated as 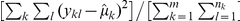. After 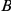 rounds of simulations and estimations (including the selection of 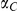), it is also statistically interesting to understand the estimation mean squared error (MSE), bias and variance at each time point. The time-point-wise mean squared error, bias and variance (for the 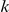-th time point, 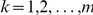) are calculated as: 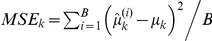; 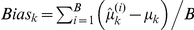; 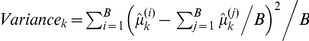. Notice that the denominator for 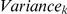 is 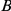 instead of 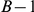 such that 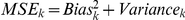.

## Results

### Simulation studies

We consider four simple scenarios to simulate time-course data: (1) 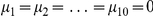, 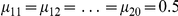, 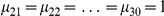, 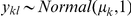; (2) 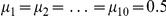, 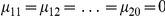, 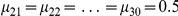, 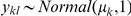; (3) 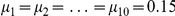, 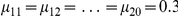, 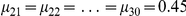, 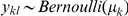; (4) 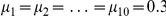, 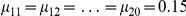, 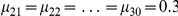, 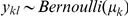. For each scenario, the number of observations is 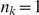, 10, or 100 at every time point. For each simulated data set, we consider twelve different values of 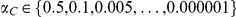. Two-tailed tests are used so that no monotonic changes are assumed. Since we know the true parameters for simulations, we choose the 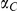 value that minimizes the overall mean squared error (MSE). This makes our simulation study computationally feasible since it is difficult to run the cross-validation procedure for many simulation repetitions. Then, for each round of simulation, we obtain the “optimized” overall MSE and the corresponding 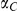 for each of the three algorithms: the global optimization algorithm (our dynamic programming algorithm) and two approximation algorithms (the recursive combination algorithm and the recursive partition algorithm). After 1000 repetitions, we compare the boxplots of these two results. A lower overall MSE is obviously preferred. But a lower 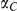 value can also be preferred since the detected changes will be statistically more significant. (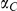 can be considered as a smoothing parameter if we do not have a pre-specified value for it. However, it also defines the significant level for the test between any two adjacent blocks. Then, a smaller 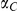 indicates a more significant testing results and it is more preferred.) Therefore, a lower boxplot means a better performance for both results.

**Figure 2:**
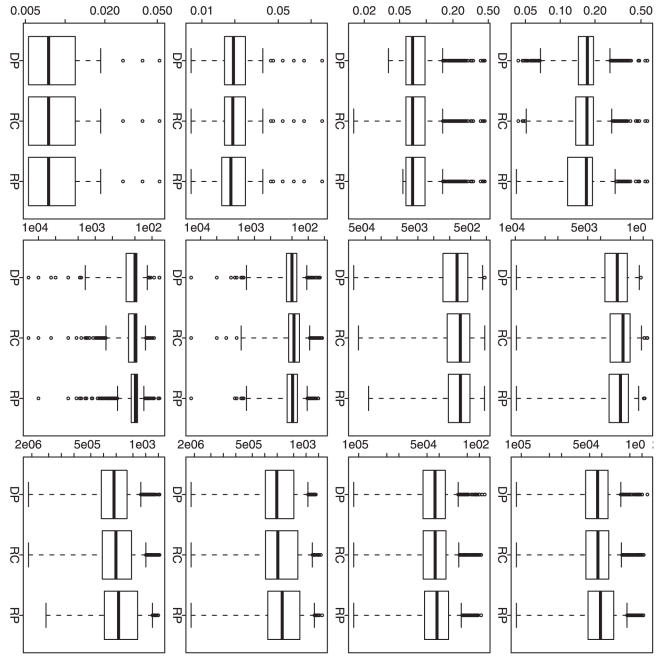
Simulation based comparison of the overall mean squared errors. All y-axes represent the overall mean squared error. DP represents our dynamic programming algorithm; RC and RP represent the recursive combination and recursive partition algorithms, respectively. The boxplots in each row (1–4) are generated from the analysis results based on the corresponding simulation scenario (1–4). The boxplots in each column (1–3) are generated from the analysis results based on different 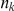 (1, 10 and 100).

For all the above four scenarios, Figures 2 and 4 shows similar patterns. When 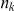 is as small as one for each time point, the approximation algorithms give a better performance in term of overall MSE, but the global optimization algorithm still gives a quite comparable performance (Figure 2); on the choice of 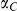, the global optimization algorithm gives a better performance and the approximation algorithms can give a comparable performance (Figure 4). When 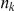 becomes larger to 10 and then to 100, we observe a clear performance improvement from the global optimization algorithm: we can achieve a clearly smaller overall MSE and also much more significant 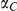 (Figures 2 and 4).

**Figure 3:**
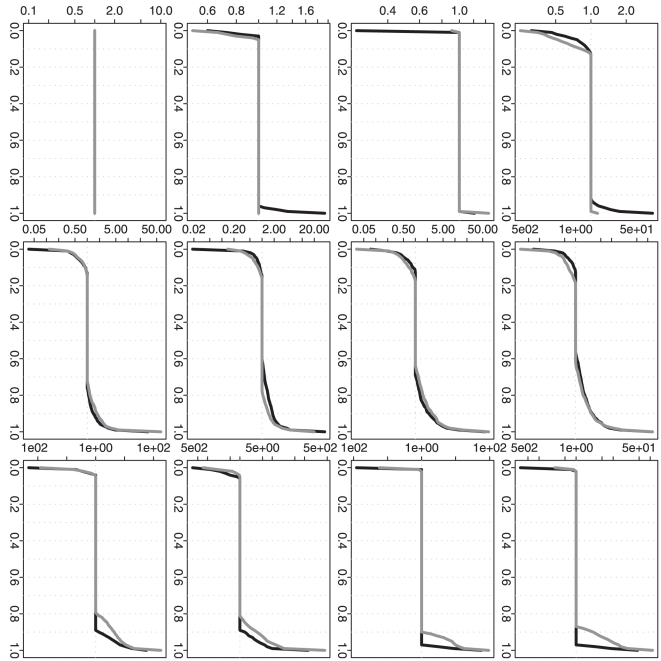
Simulation based comparison of the overall mean squared errors. All y-axes represent the quantile of relative ratio of overall MSEs given by each of the two approximation methods vs. our proposed method. All x-axes represent the values used to calculate the empirical quantiles. The black curves represent RC vs. DP and the gray curves represent RP vs. DP. (DP represents our dynamic programming algorithm; RC and RP represent the recursive combination and recursive partition algorithms, respectively.) The plots in each row (1–4) are generated from the analysis results based on the corresponding simulation scenario (1–4). The plots in each column (1–3) are generated from the analysis results based on different 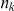 (1, 10 and 100).

**Figure 4:**
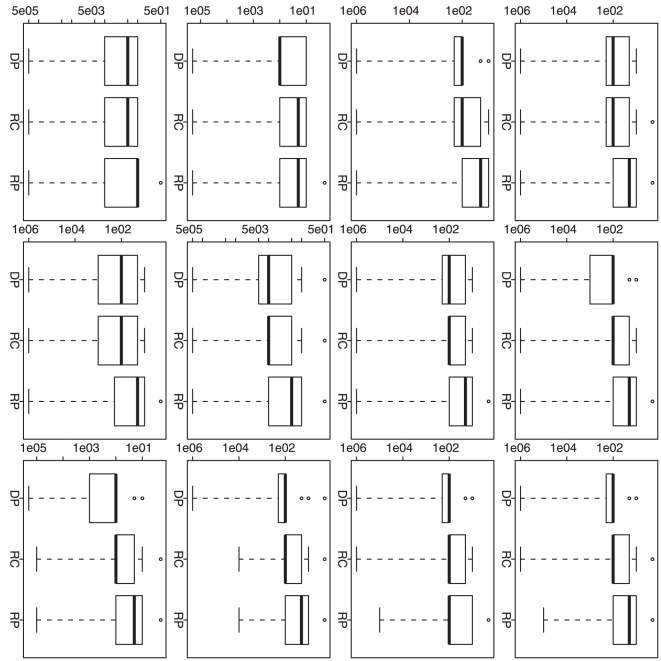
Simulation based comparison of the selected 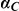. All y-axes represent the selected 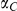. DP represents our dynamic programming algorithm; RC and RP represent the recursive combination and recursive partition algorithms, respectively. The boxplots in each row (1–4) are generated from the analysis results based on the corresponding simulation scenario (1–4). The boxplots in each column (1–3) are generated from the analysis results based on different 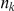 (1, 10 and 100).

**Figure 5:**
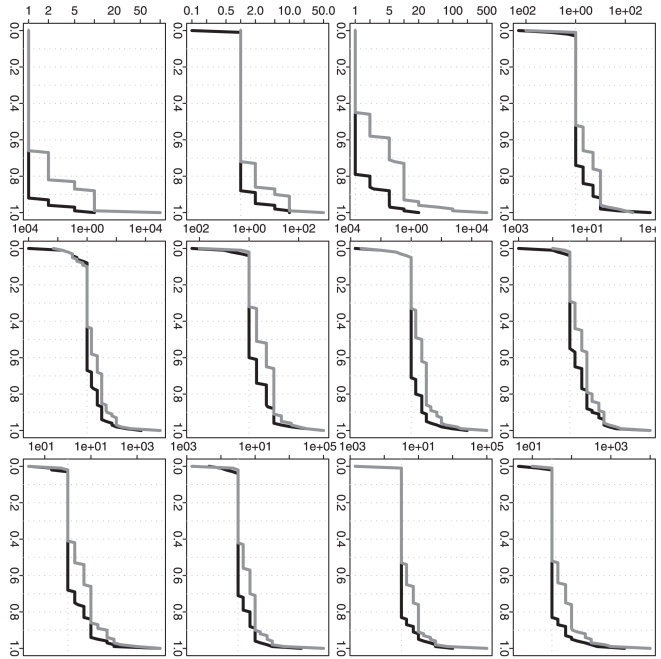
Simulation based comparison of the selected 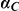. All y-axes represent the quantile of relative ratio of 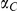
's from each of the two approximation methods vs. our proposed method. All x-axes represent the values used to calculate the empirical quantiles. The black curves represent RC vs. DP and the gray curves represent RP vs. DP. (DP represents our dynamic programming algorithm; RC and RP represent the recursive combination and recursive partition algorithms, respectively.) The plots in each row (1–4) are generated from the analysis results based on the corresponding simulation scenario (1–4). The plots in each column (1–3) are generated from the analysis results based on different 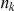 (1, 10 and 100).

To further compare the performance of three different methods, we calculate the relative ratio between two overall MSEs (or the selected 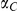
's) given by each of the two approximation methods (RC or RP) vs. our proposed method (DP). Based on 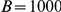 simulation repetitions, we can understand the empirical distributions of these ratios. If any ratio distribution is always no less than one, then DP is absolutely a better choice. Furthermore, for a ratio distribution, If the proportion of (ratio 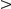
1) is clearly larger than the proportion of (ratio 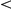
1), then DP is still a preferred choice in practice. Corresponding to Figure 2, Figure 3 further demonstrates the advantage of DP when the sample size is not relatively small. Even when the sample size is as small as one at each time point, DP still shows a quite comparable performance. For the selected 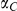, Figure 5 corresponds to Figure 4 and it also further confirms the advantage of DP. [In each plot, the proportion of (ratio 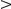
1) is cumulated from the right end although the proportion of (ratio 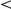
1) is cumulated from the left end.]

**Figure 6:**
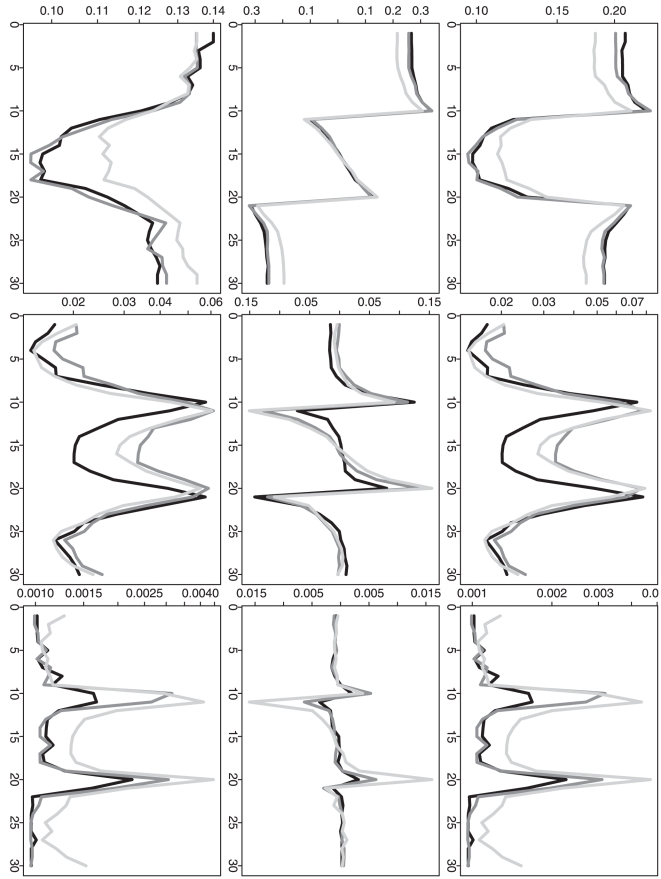
Simulation based comparison of time-point-wise MSE, bias and variance. The y-axes represent the time-point-wise MSE (upper row), bias (middle row) or variance (lower row). The x-axes represent the time point. The plots are generated from the analysis results based on the simulation scenario 1. The plots in each column (1–3) are generated from the analysis results based on different 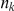 (1, 10 and 100).

**Figure 7:**
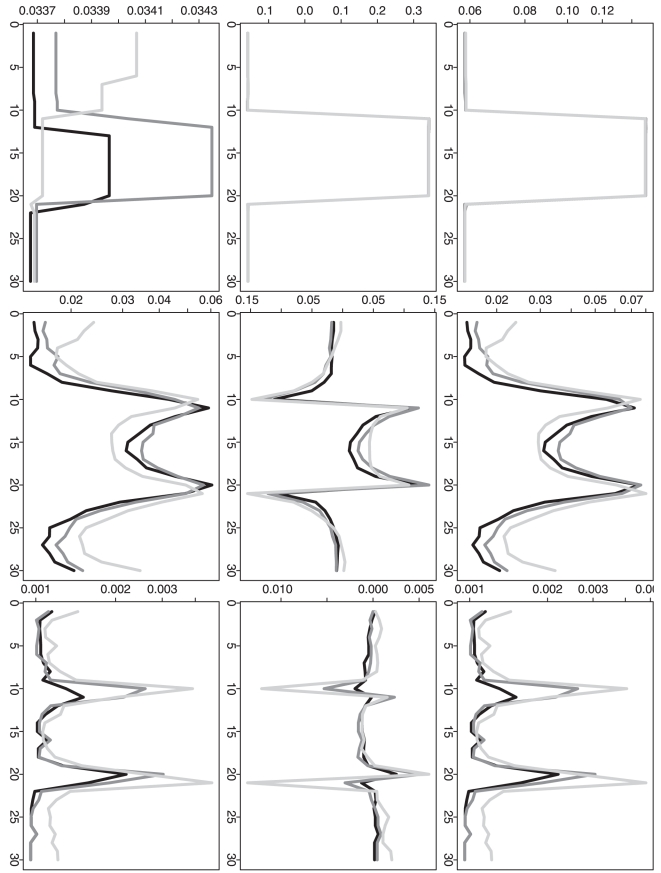
Simulation based comparison of time-point-wise MSE, bias and variance. The y-axes represent the time-point-wise MSE (upper row), bias (middle row) or variance (lower row). The x-axes represent the time point. The plots are generated from the analysis results based on the simulation scenario 2. The plots in each column (1–3) are generated from the analysis results based on different 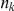 (1, 10 and 100).

**Figure 8:**
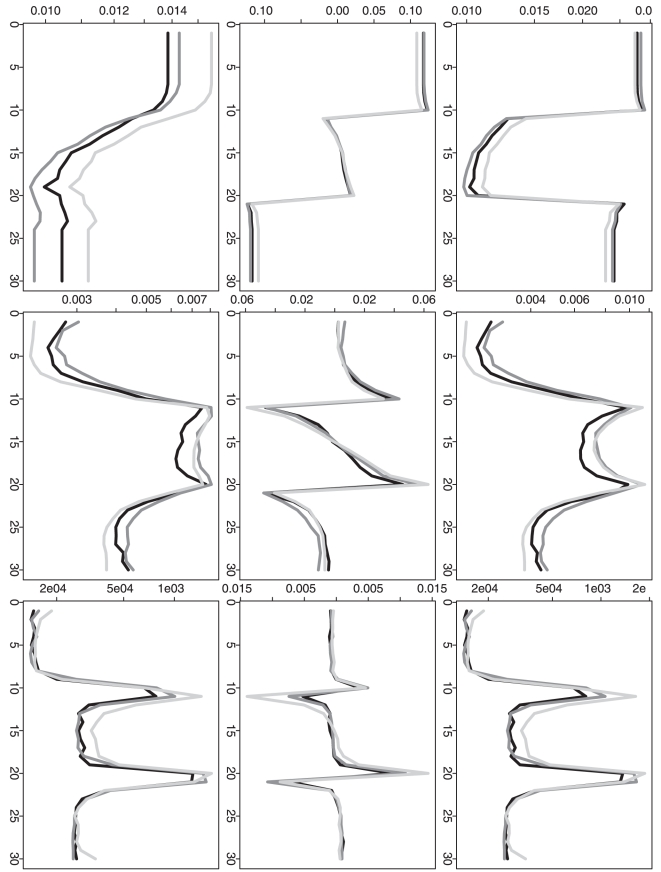
Simulation based comparison of time-point-wise MSE, bias and variance. The y-axes represent the time-point-wise MSE (upper row), bias (middle row) or variance (lower row). The x-axes represent the time point. The plots are generated from the analysis results based on the simulation scenario 3. The plots in each column (1–3) are generated from the analysis results based on different 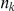 (1, 10 and 100).

**Figure 9:**
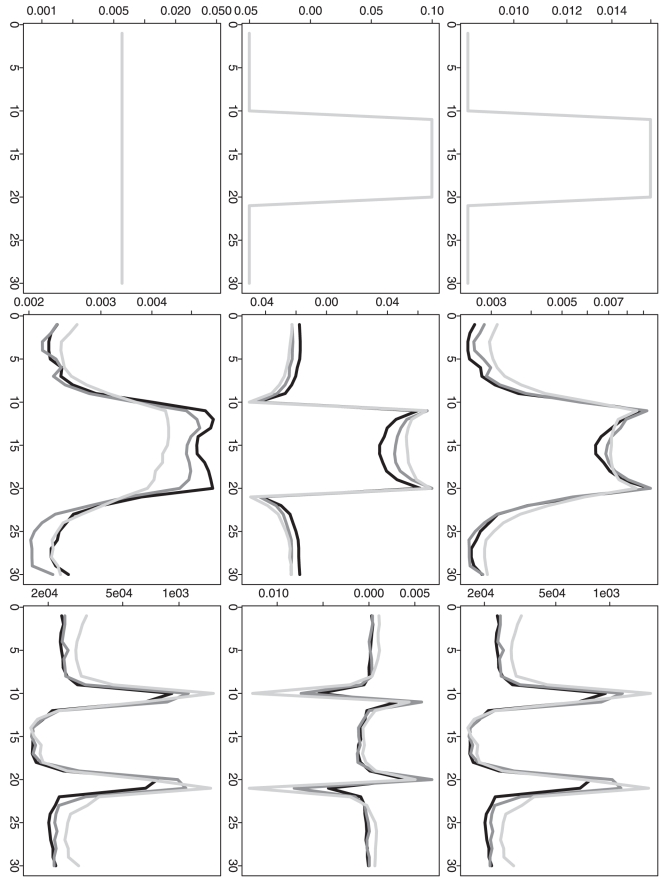
Simulation based comparison of time-point-wise MSE, bias and variance. The y-axes represent the time-point-wise MSE (upper row), bias (middle row) or variance (lower row). The x-axes represent the time point. The plots are generated from the analysis results based on the simulation scenario 4. The plots in each column (1–3) are generated from the analysis results based on different 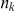 (1, 10 and 100).

In addition to the overall performance based on the overall MSE and the selected 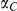, it is also statistically interesting to understand the estimation mean squared error, bias and variance at each time point. The time-point-wise mean squared error (MSE), bias and variance (for the 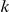-th time point, 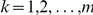) are shown in Figures 6, 7, 8, 9 for three different methods. For the time-point-wise MSE, even when the sample size is relatively small (one observation at each time point), our proposed method (DP) still shows an overall comparable performance when it is compared to the two approximation methods (RC or RP). As the sample size is increased, its time-point-wise MSEs become overall comparably lower and lower. For the time-point-wise bias, when sample size is relatively small (one at each time point), DP shows an overall worse performance in the simulation scenarios 1 and 3 but it still shows an overall comparable performance in the simulation scenarios 2 and 4. As the sample size is increased, its biases become overall comparably lower and lower (i.e. closer to the zero y-axis value). For the time-point-wise variance, DP always shows an overall comparable performance. (When the sample size is as small as one at each time point, the estimated time-point-wise means are almost all constants from all three different methods in the simulation scenarios 2; then the corresponding time-point-wise variance patterns are relatively flat. For the same sample size, the estimated time-point-wise means are actually all constants from all three different methods in the simulation scenarios 4; then the corresponding time-point-wise variances are actually constant across the whole time period. This also explains the relatively regular patterns of the corresponding time-point-wise MSE and bias.)

### Applications

For applications, we consider different univariate analysis scenarios for the well-known Pima Indian diabetes data [22]. The data set contains a binary variable for the indication of diabetes and three continuous variables for the plasma glucose concentration at 2 hours in an oral glucose tolerance test (OGTT), BMI and age (other variables in this data set are not considered in our study). Our proposed method allows us to detect the multiple change-points for diabetes indication vs. OGTT, BMI or age (analysis for a binary response), and also OGTT vs. BMI or age (analysis for a continuous response). To reduce the computation burden, the observed OGTT values are rounded to the nearest 5 units and the observed BMI values are rounded to the nearest 1 unit when any of these two variables is considered as a “time” variable. 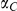 is chosen from the finite set of values 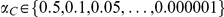 to minimize the leave-one-out cross-validation (LOO-CV) prediction error. Two-tailed tests are used so that no monotonic changes are assumed. Again, we compare three algorithms: the global optimization algorithm (our dynamic programming algorithm) and two approximation algorithms (the recursive combination algorithm and the recursive partition algorithm).

For each analysis scenario and each algorithm, Table 1 gives the “optimized” LOO-CV error and the corresponding 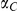. The global optimization algorithm always chooses a highly significant 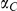 while the approximation algorithms sometimes choose a relatively large value of 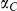. In term of prediction error, the global optimization algorithm achieves the best performance in four out of five scenarios. Although the approximation algorithms give the best prediction error for the analysis of OGTT vs. BMI, the global optimization algorithm only gives a slightly worse prediction error.

**Table 1:** Comparison of the leave-one-out cross-validation errors and the selected 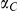
's.

	DP		RC		RP	
Analysis scenario	error	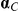	error	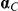	error	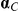
Diabetes vs. OGTT	129.85	0.00001	133.68	0.0001	129.85	0.00001
Diabetes vs. BMI	151.34	0.00001	155.92	0.1	156.96	0.05
Diabetes vs. Age	155.91	0.0001	156.88	0.001	156.53	0.1
OGTT vs. BMI	44.88	0.001	44.79	0.005	44.79	0.01
OGTT vs. Age	44.22	0.00005	44.51	0.005	44.85	0.01

DP represents our dynamic programming algorithm, RC and RP represent the recursive combination and recursive partition algorithms, respectively.

**Figure 10:**
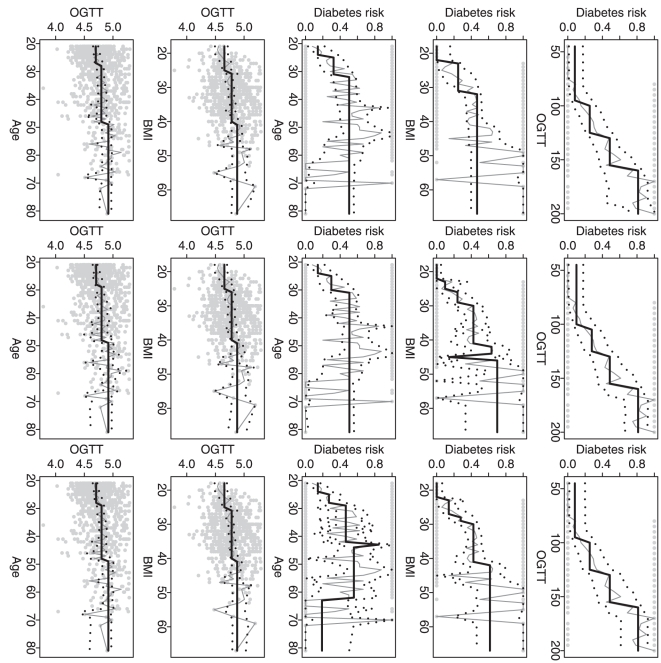
Comparison of detected change-points. The plots in each row (1–5) are generated from the results based on different analysis scenarios (as shown in the axis labels). The plots in each column (1–3) are generated from the results based on different algorithms (DP, RC and RP). In each plot, the black solid curve represents the estimated proportions/means and the black dotted curves represent the estimated 95% confidence intervals. The gray solid curve represents the estimates only based the observations at each time point. The gray dots represent the observed data.

Figure 10 shows the identified change-points for all five analysis scenarios and also all three algorithms. The global optimization algorithm always gives stable change patterns while the approximation algorithms sometimes give abrupt drops or jumps. The change patterns fitted by the global optimization algorithm are all increasing and this is practically meaningful. For example, the analysis result for diabetes indication vs. OGTT suggests significant increasing risks of diabetes when OGTT values are increased to 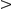
100, 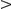
130 and 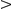
160; the analysis result for diabetes indication vs. BMI suggest significant increasing risks of diabetes when BMI values are increased to 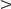
23 and 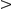
32; and the analysis results for diabetes indication vs. age suggest significant increasing risks of diabetes when age values are 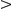
25 and 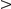
32. Since OGTT is an important predictor for diabetes, it is also interesting to understand its changes over different BMI or age intervals. Figure 10 shows increasing patterns for OGTT vs. BMI and OGTT vs. age. The change-points identified by the global optimization algorithm are 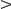
25 and 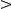
40 for OGTT vs. BMI, and 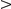
27 and 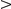
48 for OGTT vs. age.

## Discussion

The advantage of our proposed method is that the maximum likelihood estimation can be achieved during the partition of a time course. Furthermore, the method is simple and the interpretation of estimation results is clear. Based on our knowledge, the modified dynamic programming algorithm proposed in this study is novel. Although the algorithm requires more computing time than does the traditional dynamic programming algorithm, it is still practically feasible with the current computing power for general scientists. We have demonstrated the use of our algorithm for normal and binary response variables. It is also feasible to modify the algorithm for other types of response variables. Furthermore, it is interesting to explore whether there are better approaches to the choice of 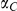. These research topics will be pursued in the near future.

One disadvantage of our method, which is actually a common disadvantage for general nonparametric methods, is that a relatively large sample size is required in order to achieve a satisfactory detection power. (This is consistent with the results in Figure 2.) For our method, we would require a relatively long time course, or a relatively large number of observations at each time point. In our simulation and application studies, we choose to analyze the data sets with relatively long time courses since this well illustrates the advantage of our method.

Our method may also be useful for the current wealthy collection of genomics data. For example, we can apply the method to array-based comparative genomics hybridization (aCGH) data and identify chromosomal aberration/alteration regions [6]; we can also apply the method to certain time-course microarray gene expression data and cluster different genes based on their changing pattern across the time course. However, a powerful computer workstation/cluster may be necessary due to the relatively high computing burden from our method.

## Supporting Information

File S1 (PDF)
