# Supplementary material for: On the Adaptive Partition Approach to the Detection of Multiple Change-Points
Source: PLoS One. 2011 May 24;6(5):e19754. doi: 10.1371/journal.pone.0019754 (PMC3101215; doi:10.1371/journal.pone.0019754)
Supplement: File S1 — (PDF) [file pone.0019754.s001.pdf]

Supporting Information S1

Yinglei Lai*
Department of Statistics and Biostatistics Center, The George Washington University, Washington, D.C., U.S.A.
* E-mail: Corresponding ylai@gwu.edu

# Mathematical proof.

We give the mathematical proof for Theorem 2. The theorem holds for the normal response data mentioned in Section 2.1 as well as the binary response data mentioned in Section 2.6. We divide the proof into several steps as lemmas. Except Lemma 3, most mathematical derivations are common for these two types of response data. The proof of Lemma 3 is presented separately for the normal and binary response data. Notice that $\alpha_C > 0$ as stated in Theorem 2.

Consider the partition of time points that is consistent with the structure of the underlying true population means: $[x_{m_0+1}, \ldots, x_{m_1}]$, ..., $[x_{m_1+m_2+\ldots+m_{g-1}+1}, \ldots, x_{m_1+m_2+\ldots+m_g}]$, where $m_0 + 1 = 1$ and $m_1 + m_2 + \ldots + m_g = m$. Let $\bar{y}_i = \sum_{j=1}^{n_i} y_{ij}/n_i$, and

$$\tilde{\mu}_i = \frac{\sum_{y_{ij}\in A} y_{ij}}{\text{Number of observations in } A},$$

where set A contains all the response observations y_{ij} in the partitioned time interval containing x_i. Furthermore, let $\hat{\mu}_i$ be the estimated population mean from our proposed algorithm.

Lemma 1. If $\lim_{n\to\infty} X_n \to a$ in probability, and $\lim_{n\to\infty} Y_n \to b$ in probability, then $\lim_{n\to\infty} X_n+Y_n \to a + b$ in probability.
Proof. This is just a basic result from fundamental probability theories.

Lemma 2. For any time point x_i, $\lim_{n_i\to\infty} |\bar{y}_i - \tilde{\mu}_i| \to 0$ in probability.
Proof. Notice that both $\bar{y}_i$ and $\tilde{\mu}_i$ are the sample means of μ_i: $\bar{y}_i$ is based on the single time point x_i, and $\tilde{\mu}_i$ is based on more time points. Therefore, we have $\lim_{n_i\to\infty} \bar{y}_i \to \mu_i$ in probability, and $\lim_{n_i\to\infty} \tilde{\mu}_i \to \mu_i$ in probability.

Together with Lemma 1, we have the following result.

$$\begin{aligned} \lim_{n_i\to\infty} |\bar{y}_i - \tilde{\mu}_i| &= \lim_{n_i\to\infty} |\bar{y}_i - \mu_i + \mu_i - \tilde{\mu}_i| \\ \leq \quad & \lim_{n_i\to\infty} |\bar{y}_i - \mu| + \lim_{n_i\to\infty} |\mu - \tilde{\mu}_i| \to 0 \text{ in probability.} \end{aligned}$$

Lemma 3. Let $n = \sum_i n_i$. Assume that $0 < a < n_i/n < b < 1$ and $\{[x_{m_0+1}, \ldots, x_{m_1}], \ldots, [x_{m_1+m_2+\ldots+m_{g-1}+1}, \ldots, x_{m_1+m_2+\ldots+m_g}]\}$ is a feasible partition of time points, for any time point x_i, we have $\lim_{n_i\to\infty} |\bar{y}_i - \hat{\mu}_i| \to 0$ in probability.
Proof for normal response data. Our proposed algorithm estimates the population means by minimizing the sum of squared errors (SSE) in a restricted space. Also notice that the smallest SSE is $\sum_{i=1}^{m}\sum_{j=1}^{n_i}(y_{ij} - \bar{y}_i)^2$, and $\{\tilde{\mu}_i\}$ are the estimated population means when $\{[x_{m_0+1}, \ldots, x_{m_1}], \ldots, [x_{m_1+m_2+\ldots+m_{g-1}+1}, \ldots, x_{m_1+m_2+\ldots+m_g}]\}$ is a feasible partition of time points. Then, we have

$$
\begin{aligned}
&\sum_{i=1}^{m}\sum_{j=1}^{n_i}(y_{ij}-\bar{y}_i)^2 \le \sum_{i=1}^{m}\sum_{j=1}^{n_i}(y_{ij}-\hat{\mu}_i)^2 \le \sum_{i=1}^{m}\sum_{j=1}^{n_i}(y_{ij}-\tilde{\mu}_i)^2 \\
\Rightarrow\quad & 0 \le \sum_{i=1}^{m}\sum_{j=1}^{n_i}(y_{ij}-\hat{\mu}_i)^2 - \sum_{i=1}^{m}\sum_{j=1}^{n_i}(y_{ij}-\bar{y}_i)^2 \le \sum_{i=1}^{m}\sum_{j=1}^{n_i}(y_{ij}-\tilde{\mu}_i)^2 - \sum_{i=1}^{m}\sum_{j=1}^{n_i}(y_{ij}-\bar{y}_i)^2 \\
\Rightarrow\quad & 0 \le \sum_{i=1}^{m}\sum_{j=1}^{n_i}(\bar{y}_i-\hat{\mu}_i)^2 \le \sum_{i=1}^{m}\sum_{j=1}^{n_i}(\bar{y}_i-\tilde{\mu}_i)^2 \\
\Rightarrow\quad & 0 \le \sum_{i=1}^{m} n_i(\bar{y}_i-\hat{\mu}_i)^2 \le \sum_{i=1}^{m} n_i(\bar{y}_i-\tilde{\mu}_i)^2. \\
\Rightarrow\quad & 0 \le \sum_{i=1}^{m}(n_i/n)(\bar{y}_i-\hat{\mu}_i)^2 \le \sum_{i=1}^{m}(n_i/n)(\bar{y}_i-\tilde{\mu}_i)^2.
\end{aligned}
$$

Since $\lim_{n_i\to\infty}|\bar{y}_i-\tilde{\mu}_i| \to 0$ in probability, then $\lim_{n_i\to\infty}(\bar{y}_i-\tilde{\mu}_i)^2 \to 0$ in probability. Furthermore, we have assumed that $0 < a < n_i/n < b < 1$, then $\lim_{n\to\infty}\sum_{i=1}^{m}(n_i/n)\ (\bar{y}_i-\tilde{\mu}_i)^2 \to 0$ in probability. Therefore, $\lim_{n\to\infty}\sum_{i=1}^{m}(n_i/n)(\bar{y}_i-\hat{\mu}_i)^2 \to 0$ in probability. Again, with the assumption that $0 < a < n_i/n < b < 1$, we have $\lim_{n_i\to\infty}|\bar{y}_i-\hat{\mu}_i| \to 0$ in probability.

Proof for binary response data. Our proposed algorithm estimates the population proportions by maximizing the log-likelihood in a restricted space. The log-likelihood function formula is

$$
\begin{aligned}
l(\{\mu_i\}) &= \sum_{ij}[y_{ij}\log(\mu_i)+(1-y_{ij})\log(1-\mu_i)] \\
&= \sum_i n_i[\bar{y}_i\log(\mu_i)+(1-\bar{y}_i)\log(1-\mu_i)] = \sum_i n_i l_i(\mu_i),
\end{aligned}
$$

where we define $l_i(\mu_i) = \bar{y}_i\log(\mu_i)+(1-\bar{y}_i)\log(1-\mu_i)$.

It is obvious that the largest log-likelihood can be achieved when $\mu_i = \bar{y}_i$. Then, based on our algorithm, we have $l(\{\bar{y}_i\}) \ge l(\{\hat{\mu}_i\}) \ge l(\{\tilde{\mu}_i\})$. This is equivalent to

$$
\begin{aligned}
& 0 \le l(\{\bar{y}_i\}) - l(\{\hat{\mu}_i\}) \le l(\{\bar{y}_i\}) - l(\{\tilde{\mu}_i\}) \\
\Leftrightarrow\quad & 0 \le \sum_i n_i[l_i(\bar{y}_i)-l_i(\hat{\mu}_i)] \le \sum_i n_i[l_i(\bar{y}_i)-l_i(\tilde{\mu}_i)] \\
\Leftrightarrow\quad & 0 \le \sum_i \frac{n_i}{n}[l_i(\bar{y}_i)-l_i(\hat{\mu}_i)] \le \sum_i \frac{n_i}{n}[l_i(\bar{y}_i)-l_i(\tilde{\mu}_i)].
\end{aligned}
$$

Based on Lemma 2, also notice that $0 \le \frac{n_i}{n}, \bar{y}_i, 1-\bar{y}_i \le 1$ we have

$$
\begin{aligned}
&\sum_i \frac{n_i}{n}[l_i(\bar{y}_i)-l_i(\tilde{\mu}_i)] \\
&= \sum_i \frac{n_i}{n}\{\bar{y}_i[\log(\bar{y}_i)-\log(\tilde{\mu}_i)]+(1-\bar{y}_i)[\log(1-\bar{y}_i)-\log(1-\tilde{\mu}_i)]\} \\
&\le \sum_i\{|\log(\bar{y}_i)-\log(\tilde{\mu}_i)|+|\log(1-\bar{y}_i)-\log(1-\tilde{\mu}_i)|\} \\
&\to 0 \text{ in probability .}
\end{aligned}
$$

Then, we have

$$
\sum_i \frac{n_i}{n}[l_i(\bar{y}_i)-l_i(\hat{\mu}_i)] \to 0 \text{ in probability.}
$$

Since $0 < a < n_i/n < b < 1$ and $l_i(\bar{y}_i) \geq l_i(\hat{\mu}_i)$ $(l_i(\bar{y}_i) - l_i(\hat{\mu}_i) \geq 0)$, we have

$$l_i(\bar{y}_i) - l_i(\hat{\mu}_i) \to 0 \text{ in probability.}$$

At this point, it is less obvious that $\bar{y}_i - \hat{\mu}_i \to 0$ in probability and we prove this by contradiction. Assume that $\hat{\mu}_i$ does not converge to $\bar{y}_i$ in probability, then for any given $\epsilon_1 > 0$, there is $\delta_1 > 0$ and we can always find an integer n_i (no matter how large it is), such that $\Pr(|\hat{\mu}_i - \bar{y}_i| \geq \epsilon_1) \geq \delta_1$.

Since y_{ij} follows a Bernoulli distribution with parameter μ_i, we have $\bar{y}_i \to \mu_i$ in probability. Then, given any $\epsilon_2 > 0$ and $\delta_1 > \delta_2 > 0$, we can find an integer N_i such that $\Pr(|\bar{y}_i - \mu_i| < \epsilon_2) > 1 - \delta_2$ when $n_i > N_i$.

Now, define the region $A = \{(\bar{y}_i, \hat{\mu}_i) : |\hat{\mu}_i - \bar{y}_i| \geq \epsilon_1 \text{ and } |\bar{y}_i - \mu_i| < \epsilon_2\}$, and let $m = \min_{(\bar{y}_i, \hat{\mu}_i) \in A}[l_i(\bar{y}_i) - l_i(\hat{\mu}_i)]$. (Notice that $m > 0$ based on the definition of function l_i; $m = 0$ only when at least one pair of $(\bar{y}_i, \hat{\mu}_i = \bar{y}_i) \in A$, which contradicts the definition of region A.) Then, when $n_i > N_i$,

$$\begin{aligned}
&\Pr(|l_i(\bar{y}_i) - l_i(\hat{\mu}_i)| \geq m) \\
&= \Pr(|l_i(\bar{y}_i) - l_i(\hat{\mu}_i)| \geq m | (\bar{y}_i, \hat{\mu}_i) \in A) \Pr((\bar{y}_i, \hat{\mu}_i) \in A) \\
&+ \Pr(|l_i(\bar{y}_i) - l_i(\hat{\mu}_i)| \geq m | (\bar{y}_i, \hat{\mu}_i) \notin A) \Pr((\bar{y}_i, \hat{\mu}_i) \notin A) \\
&\geq \Pr(|l_i(\bar{y}_i - l_i(\hat{\mu}_i)| \geq m | (\bar{y}_i, \hat{\mu}_i) \in A) \Pr((\bar{y}_i, \hat{\mu}_i) \in A) \\
&= 1 \times \Pr((\bar{y}_i, \hat{\mu}_i) \in A) \text{ based on the definition of } m \\
&= \Pr(|\hat{\mu}_i - \bar{y}_i| \geq \epsilon_1 \text{ and } |\bar{y}_i - \mu_i| < \epsilon_2) \\
&\geq \Pr(|\hat{\mu}_i - \bar{y}_i| \geq \epsilon_1) - \delta_2.
\end{aligned}$$

The last inequality holds since

$$\begin{aligned}
&\Pr(|\hat{\mu}_i - \bar{y}_i| \geq \epsilon_1) \\
&= \Pr(|\hat{\mu}_i - \bar{y}_i| \geq \epsilon_1 \text{ and } |\bar{y}_i - \mu_i| < \epsilon_2) + \Pr(|\hat{\mu}_i - \bar{y}_i| \geq \epsilon_1 \text{ and } |\bar{y}_i - \mu_i| \geq \epsilon_2) \\
&\leq \Pr(|\hat{\mu}_i - \bar{y}_i| \geq \epsilon_1 \text{ and } |\bar{y}_i - \mu_i| < \epsilon_2) + \Pr(|\bar{y}_i - \mu_i| \geq \epsilon_2) \\
&\leq \Pr(|\hat{\mu}_i - \bar{y}_i| \geq \epsilon_1 \text{ and } |\bar{y}_i - \mu_i| < \epsilon_2) + \delta_2.
\end{aligned}$$

However, based on our assumption, we can always find an integer $n_i > N_i$ (no matter how large it is) such that $\Pr(|\hat{\mu}_i - \bar{y}_i| \geq \epsilon_1) \geq \delta_1$. Then, we have

$$\Pr(|l_i(\bar{y}_i) - l_i(\hat{\mu}_i)| \geq m) \geq \delta_1 - \delta_2 > 0.$$

This contradicts our previous results that $l_i(\bar{y}_i) - l_i(\hat{\mu}_i) \to 0$ in probability. Therefore, we must have $\bar{y}_i - \hat{\mu}_i \to 0$ in probability.

Lemma 4. Under the assumptions of Lemma 3, we have $\lim_{n_i \to \infty} \hat{\mu}_i \to \mu_i$ in probability.
Proof. Based on results of Lemmas 1 and 3 and the proof of Lemma 2, we have

$$\lim_{n_i \to \infty} |\hat{\mu}_i - \mu_i| \leq \lim_{n_i \to \infty} |\hat{\mu}_i - \bar{y}_i| + \lim_{n_i \to \infty} |\bar{y}_i - \mu_i| \to 0 \text{ in probability.}$$

Lemma 5. Let $n = \sum_i n_i$. Assume that $0 < a < n_i/n < b < 1$, then $\lim_{n \to \infty} \Pr(\{\ [x_{m_0+1}, \ldots, x_{m_1}],$ $\ldots, [x_{m_1+m_2+\ldots+m_{g-1}+1}, \ldots, x_{m_1+m_2+\ldots+m_g}]\ \}$ is not a feasible partition of time points$) \to 0$.
Proof. The probability is equivalent to Pr(at least two adjacent $\{[x_{m_0+1}, \ldots, x_{m_1}], \ldots, [x_{m_1+m_2+\ldots+m_{g-1}+1},$ $\ldots, x_{m_1+m_2+\ldots+m_g}]\}$ have the test p-value larger than the given α_C). This probability is less than $(m_g - 1) \times \max_{k \in \{1,2,\ldots,m_g-1\}}\{$ Pr($[x_{m_1+m_2+\ldots+m_{k-1}+1}, \ldots, x_{m_1+m_2+\ldots+m_k}]$ and $[x_{m_1+m_2+\ldots+m_k+1}, \ldots,$ $x_{m_1+m_2+\ldots+m_{k+1}}]$ have the test p-value larger than the given α_C)$\}$, which goes to zero as $n_i \to \infty$. (When

$n \to \infty$, each $n_i \to \infty$ based on the assumption.) Therefore,
Pr({ $[x_{m_0+1}, \ldots, x_{m_1}]$, $\ldots$, $[x_{m_1+m_2+\ldots+m_{g-1}+1}, \ldots, x_{m_1+m_2+\ldots+m_g}]$ } is not a feasible partition of time points) $\to 0$ as $n_i \to \infty$

Theorem 2. Let $n = \sum_i n_i$. Assume that $0 < a < n_i/n < b < 1$ and $\alpha_C > 0$. Then, for any time point x_i, we have $\lim_{n_i \to \infty} \hat{\mu}_i \to \mu_i$ in probability, or our proposed algorithm provides consistent estimates for $\{\mu_i : i = 1, 2, \ldots, m\}$. Furthermore, we also have $\lim_{n \to \infty} \hat{\sigma}^2 \to \sigma^2$ in probability, or our proposed algorithm also provides a consistent estimate for σ^2.
Proof. This theorem is derived based on Lemmas 1-5. For simplicity, we use "$\{\tilde{\mu}_i\}$ feasible" to denote that $\{[x_{m_0+1}, \ldots, x_{m_1}], \ldots, [x_{m_1+m_2+\ldots+m_{g-1}+1}, \ldots, x_{m_1+m_2+\ldots+m_g}]\}$ is a feasible partition of time points, and similarly for the notation "$\{\tilde{\mu}_i\}$ not feasible". For any given $\epsilon > 0$,

$$\begin{aligned} & \Pr(|\hat{\mu}_i - \mu_i| \geq \epsilon) \\ = \; & \Pr(|\hat{\mu}_i - \mu_i| \geq \epsilon | \{\tilde{\mu}_i\} \text{ feasible}) \times \Pr(\{\tilde{\mu}_i\} \text{ feasible}) \\ + \; & \Pr(|\hat{\mu}_i - \mu_i| \geq \epsilon | \{\tilde{\mu}_i\} \text{ not feasible}) \times \Pr(\{\tilde{\mu}_i\} \text{ not feasible}) \\ \leq \; & \Pr(|\hat{\mu}_i - \mu_i| \geq \epsilon | \{\tilde{\mu}_i\} \text{ feasible}) + \Pr(\{\tilde{\mu}_i\} \text{ not feasible}) \\ \to \; & 0 \text{ as } n \to \infty. \end{aligned}$$

Therefore, we have $\lim_{n_i \to \infty} \hat{\mu}_i \to \mu_i$ in probability.

For the convergence of $\hat{\sigma}^2$, we first define and consider $\hat{\sigma}_i^2 = \sum_{j=1}^{n_i} (y_{ij} - \hat{\mu}_i)^2/(n_i - 1)$. Let $\tilde{\sigma}_i^2 = \sum_{j=1}^{n_i} (y_{ij} - \bar{y}_i)^2/(n_i - 1)$. $\hat{\sigma}_i^2$ and $\tilde{\sigma}_i^2$ are closely related.

$$\begin{aligned} \hat{\sigma}_i^2 &= \sum_{j=1}^{n_i} (y_{ij} - \bar{y}_i + \bar{y}_i - \hat{\mu}_i)^2/(n_i - 1) \\ &= \tilde{\sigma}_i^2 + (\bar{y}_i - \hat{\mu}_i)^2 n_i/(n_i - 1). \end{aligned}$$

Since $\tilde{\sigma}_i^2 - \sigma^2 \to 0$ in probability [1] and $\bar{y}_i - \hat{\mu}_i \to 0$ by Lemma 3, we then have $\hat{\sigma}_i^2 - \sigma^2 \to 0$ in probability [1].

Finally, we notice that

$$\hat{\sigma}^2 = \sum_{i=1}^{m} \sum_{j=1}^{n_i} (y_{ij} - \hat{\mu}_i)^2 / (\sum_{i=1}^{m} n_i - 1) = \sum_{i=1}^{m} \frac{\frac{n_i}{n} - \frac{1}{n}}{1 - \frac{1}{n}} \hat{\sigma}_i^2.$$

Therefore, we have $\lim_{n \to \infty} \hat{\sigma}^2 \to \sigma^2$ in probability.

References

1. Casella G, Berger RL (2002) Statistical inference, 2nd edition. Duxbury.
